# Association between financial hardship and psychological burden and the role of social and mental health support: An observational study

**DOI:** 10.1097/MD.0000000000038871

**Published:** 2024-07-12

**Authors:** Jinho Jung, Kumar Mukherjee, Mary Brown, Gelareh Sadigh

**Affiliations:** a School of Medicine, University of California, Irvine, Irvine, CA; b Department of Radiological Sciences, University of California, Irvine, Orange, CA; c School of Pharmacy, Philadelphia College of Osteopathic Medicine, Suwanee, GA; d Adena Health System, Chilicothe, OH.

**Keywords:** financial hardship, mental health support, National Health Interview Survey, prevalence, psychological burden, serious psychological distress, social support

## Abstract

We aimed to assess the association between medical financial hardship and psychological burden and the moderating role of social and mental health support. 2021 United States National Health Interview Survey was used. Financial hardship was defined as having financial worry, material hardship, or cost-related care nonadherence. Psychological burden was measured using perceived general health status, satisfaction with life, and serious psychological distress (SPD). Of 29,370 included adults, 49% experienced financial hardship in the last 12 months. Financial hardship was associated with a higher psychological burden (odds ratio [OR], 3.58; 95% confidence interval [CI], 2.43–5.47 for SPD). Eleven percent received counseling/therapy from mental health professionals, and 90% had experienced frequent social support. Frequent social support was associated with lower financial hardship (OR, 0.71; 95% CI, 0.63–0.80) and psychological burden (OR, 0.28; 95% CI, 0.19–0.42 for SPD). Previous mental health support was associated with higher financial hardship (OR,1.40; 95% CI, 1.28–1.54) and psychological burden (OR, 9.75; 95% CI, 6.97–13.94 for SPD). Those experiencing financial hardship had lower odds of SPD if they received mental health support in the last 12 months (OR, 0.57; 95% CI, 0.39–0.85). Future interventions should also focus on improving social support and mental health for patients as a way of mitigating medical financial hardship.

## 1. Introduction

In the United States (U.S.), patients commonly incur an out-of-pocket cost for their medical services, with the amount being variable depending on patients’ insurance coverage (insured vs uninsured), insurance type (e.g., public or private), and other factors such as insurance benefit design (deductible, coinsurance, annual out-of-pocket maximum).^[1]^ However, there has been an increase in patients’ out-of-pocket medical expenses in the last decade due to increased cost-sharing from the adoption of high-deductible health plans and multiple-tier co-pays, as well as the introduction of new therapies and technologies.^[2]^ Medical financial hardship arises from high medical out-of-pocket expenses and lost income due to the medical condition or its treatment.^[3,4]^ Medical financial hardship is characterized by 3 domains of financial worry, material hardship (e.g., medical debt), and cost-related care nonadherence (e.g., forgoing or delaying medical care due to cost).^[5]^ Approximately 56% of adult Americans report medical financial hardship.^[5]^

Medical financial hardship has been shown to be associated with several downstream effects such as a decrease in quality of life and perceived health status, increased anxiety, and depression.^[6–9]^ Studies have shown that medical financial hardship and mental health problems are often correlated. A meta-analysis has shown a significant relationship between medical debt (as a domain of medical financial hardship) and mental disorders.^[10]^ On the other hand, adults with serious psychological distress (SPD) have double the odds of having medical debt compared to those without.^[11]^ A growing number of interventions such as out-of-pocket payment elimination, financial education, and financial navigation have been proposed to help alleviate medical financial hardship and its consequences.^[12,13]^ Furthermore, it is important to screen for medical financial hardship periodically throughout the course of care, as medical financial hardship waxes and wanes for many individuals.^[14]^ While there are studies that investigate interventions for medical financial hardship, there is a lack of understanding of how social support or mental health support might help with medical financial hardship and the psychological burden that is caused by it.

Using a recent nationally representative survey of the U.S. general population, we aimed to measure the prevalence of medical financial hardship and assess its correlation with psychological burden controlling for receipt of counseling from a mental health professional or having frequent social or emotional support. We further assessed the moderating role of social support and mental health support on medical financial hardship and psychological burden.

## 2. Methods

### 2.1. Ethical approval

This observational study did not use any private identifiable information and thus did not constitute human subject research requiring institutional review board oversight.

### 2.2. Study participants

We identified adults aged 18 years or older from the U.S. 2021 National Health Interview Survey (NHIS). NHIS is a nationally representative, cross-sectional household interview survey of civilian noninstitutionalized U.S. population residing within the 50 states and the District of Columbia. Those with no fixed household address, active-duty military personnel and civilians living on military bases, persons in long-term care institutions, persons in correctional facilities, and U.S. nationals living in foreign countries are excluded from NHIS. Eligible individuals are interviewed throughout the year using a cluster sampling method. The majority of interviews are conducted face-to-face in respondents’ homes, but a telephone interview may also be conducted when the respondent requests a telephone interview or when road conditions or travel distances would make it difficult to schedule a personal visit.^[15]^ In 2021, the survey response rate was 50.9%.^[15]^

### 2.3. Measures and outcomes

#### 2.3.1. Medical financial hardship

Prevalence of each of the 3 domains of medical financial hardship including financial worry, material hardship, or cost-related care nonadherence was measured. Financial worry was defined as worrying about paying for medical bills in case of sickness or accident in the past 12 months. Material hardship was defined as having problems paying or being unable to pay medical bills in the past 12 months. Cost-related care nonadherence was measured as any incidence of delay or forgo of either medical care or mental health, or a change (skipping a dose, taking less medication, delaying filling a prescription) or forgo of prescription medication due to cost in the past 12 months. We further measured medical financial hardship as a binary variable with 2 categories, (a) no hardship vs (b) presence of any of the 3 domains.

#### 2.3.2. Psychological burden

Psychological burden was measured using perceived general health status, satisfaction with life, and presence of SPD in the last 30 days measured by Kessler-6 scale.^[16]^ Perceived general health status was treated as a binary variable by combining the response categories of “excellent, very good, or good” compared to “fair or poor.” Satisfaction with life was also considered as a binary variable by grouping response categories of “very satisfied or satisfied” compared to “very dissatisfied or dissatisfied.” A respondent with a Kessler-6 score of 13 or more was classified as “experiencing serious psychological distress.”^[16]^

#### 2.3.3. Social and mental health support

Frequency of receipt of social and emotional support was measured by asking “How often do you get the social and emotional support you need?.” Participants were categorized as “Always, usually, or sometimes” versus “rarely or never.” Receipt of counseling or therapy from mental health professionals such as psychiatrists, psychologists, psychiatric nurses, or clinical social workers in the past 12 months was measured as a binary variable (yes/no).

#### 2.3.4. Sociodemographic and comorbidities

Sociodemographic variables such as age, race, sex, family income, insurance status, marital status, and number of comorbidities (hypertension, hyperlipidemia, dementia, chronic obstructive pulmonary disease, emphysema, chronic bronchitis, diabetes, history of heart attack, kidney disease, asthma, epilepsy, and cancer) were extracted from the survey results.

### 2.4. Statistical analysis

Descriptive statistics were used to report frequencies and percentages for categorical variables. Sampling weights were used to produce representative national estimates. Multivariable logistic regression models were used to assess the factors associated with medical financial hardship, as well as the correlation between medical financial hardship and psychological burden controlling for sociodemographic factors, number of comorbidities, and frequent receipt of social support or prior receipt of mental health support in the last 12 months. A complete case analysis was performed. We used SAS version 14.3 for analysis and statistical significance was defined as *P*-value <.05.

## 3. Results

### 3.1. Study participants

Our analytical sample included 29,370 adults (weighted N = 252,228,317). Weighted descriptive statistics of the sample are provided in Table 1. The mean age of the cohort was 52 years (standard deviation 18.3) with 22% having at least 65 years of age. In the weighted sample, the majority were female (52%) and were of non-Hispanic White racial and ethnic background (63%). Over one-fourth (27%) of adults had a family income <200% of the poverty threshold and 59% were married or were living with a partner. A total of 61.9% had private insurance and 9.9% were uninsured.

**Table 1:** Sociodemographic characteristics of sample (weighted frequency = 252,228,317).

	Weighted frequency, n (%)*
Demographic profile	
*Age*	
18–40 years	98,085,748 (38.89)
41–64 years	97,803,804 (38.78)
65–85 years	55,676,249 (22.07)
Others†	662,517 (0.26)
*Race, n (%*)	
Non-Hispanic white only	158,416,493 (62.81)
Non-Hispanic Black/African American only	29,376,606 (11.65)
Hispanic	42,764,820 (16.95)
Others single and multiple races	21,670,399 (8.59)
*Sex*	
Male	121,766,896 (48.28)
Female	130,445,015 (51.72)
Others†	16,406 (0.01)
*Family income as a percentage of poverty threshold*	
Poor (<100%)	24,914,719 (9.88)
Near poor (100% to <125%)	9777,706 (3.88)
Low income (125% to <200%)	34,404,007 (13.64)
Middle income (200% to <400%)	74,428,295 (29.51)
High income (≥400%)	108,703,590 (43.10)
*Insurance*	
Private	156,230,705 (61.94)
Medicare and dual eligible	29,899,782 (11.85)
Medicaid	27,532,751 (10.91)
Other insurance	12,577,975 (4.99)
Uninsured	24,973,682 (9.90)
Others†	1006,643 (0.40)
*Marital status*	
Married	126,877,329 (50.30)
Living with a partner	20,797,356 (8.25)
Unmarried or not living together	97,141,111 (38.51)
Others†	7412,520 (2.94)
*Number of comorbidities*	
=0	111,584,010 (44.23)
=1	66,282,922 (26.28)
≥2	73,813,804 (29.26)
Others†	547,582 (0.21)
*Experience of medical financial hardship*	
No financial hardship‡	125,021,420 (49.57)
Presence of financial hardship§	124,117,372 (49.21)
Others∥	3089,525 (1.22)
*Financial worry* ¶	
No financial worry	139,012,198 (55.11)
Presence of financial worry	112,535,891 (44.62)
Others†	680,228 (0.27)
*Material hardship* ¶	
No material hardship	225,106,458 (89.25)
Presence of material hardship	226,493,105 (10.50)
Other†	628,754 (0.25)
*Cost-related care nonadherence* ¶	
Adherent	212,070,197 (84.08)
Nonadherent	35,903,157 (14.23)
Others†	4254,963 (1.69)
*Frequency of receipt of social/emotional support*	
Always	140,764,033 (55.81)
Usually or sometimes	86,612,451 (34.34)
Rarely or never	16,742,176 (6.64)
Others†	8109,657 (3.22)
*Receipt of counseling/therapy from mental health professional in the past 12 m*	
Yes	27,630,324 (10.95)
No	220,184,301 (87.30)
Others†	4413,692 (3.22)
*Perceived general health status*	
Excellent, very good, good	217,952,711 (86.41)
Fair or poor	85,843,348 (13.55)
Others†	86,184 (0.03)
*Serious psychological distress (Kessler 6- scale*)	
Yes (score ≥ 13)	9029,995 (3.58)
No (score < 13)	236,257,225 (93.67)
Others†	6941,097 (2.75)
*Satisfaction with life*	
Very satisfied or satisfied	234,714,768 (93.06)
Very dissatisfied or dissatisfied	11,868,048 (4.71)
Others†	5645,500 (2.24)

* Weighted number and weighted percentage is reported.

† Others indicate response values reported as “Refused, Not Ascertained, Don’t Know.”

‡ No hardship is defined as^[1]^ all financial hardship domains = no; or (2) one or more financial hardship domains = no and the remainder of domains = missing.

§ Experiencing financial hardship is defined as one or more domains of financial hardship = yes.

∥ Others indicates all domains of financial hardship were missing.

¶ Financial Worry, cost-related care nonadherence, and material hardship are 3 domains constituting medical financial hardship.

### 3.2. Outcomes

#### 3.2.1. Medical financial hardship

A total of 49% of respondents experienced medical financial hardship, with 45% reporting financial worry, 11% material hardship, and 14% cost-related care nonadherence in the last 12 months.

Table 2 shows a multivariable logistic regression analysis of factors associated with medical financial hardship. Older age (OR, 0.99; 95% confidence interval [CI], 0.98–0.99), male gender compared to female (OR, 0.75; 95% CI, 0.70–0.79), income equal to or more than 200% of poverty line compared to <200% of poverty (OR, 0.57, 95% CI, 0.53–0.62), and Medicare (OR, 0.72; 95% CI, 0.66–0.79) or Medicaid (OR, 0.73; 95% CI, 0.65–0.82) as primary insurance (compared to commercial insurance) were associated with lower probability of experiencing medical financial hardship. On the other hand, participants who were non-Hispanic Black (OR, 1.18; 95% CI, 1.05–1.31) or Hispanic (OR, 1.66; 95% CI, 1.50–1.83) compared to those who were non-Hispanic White, participants who were uninsured compared to those with commercial insurance (OR, 3.51; 95% CI, 3.04–4.05), and those with one (OR, 1.32; 95% CI, 1.23–1.42) or more comorbidities (OR, 1.59; 95% CI, 1.47–1.72) compared to no comorbidity had higher probability of experiencing medical financial hardship.

**Table 2:** Multivariable logistic regression analysis of factors associated with any medical financial hardship.

Factors	OR (95% CI)	*P* value
Age	0.99 (0.98–0.99)	<.001
*Gender*		
Female	Reference	
Male	0.75 (0.70–0.79)	<.001
*Race*		
Non-Hispanic white only	Reference	
Non-Hispanic Black/African American only	1.18 (1.05–1.31)	.003
Hispanic	1.66 (1.50–1.83)	<.001
Others	1.25 (1.13–1.40)	<.001
*Income*		
Income < 200% of poverty line	Reference	
Income ≥ 200% of poverty line	0.57 (0.53–0.62)	<.001
*Insurance*		
Commercial	Reference	
Medicare	0.72 (0.66–0.79)	<.001
Medicaid	0.73 (0.65–0.82)	<.001
Other insurance	0.72 (0.63–0.82)	<.001
Uninsured	3.51 (3.04–4.05)	<.001
Number of comorbidities		
0	Reference	
1	1.32 (1.23–1.42)	<.001
≥2	1.59 (1.47–1.72)	<.001
Frequent receipt of social or emotional support	0.71 (0.63–0.80)	<.001
Receipt of counseling/therapy from mental health professional in the past 12 m	1.40 (1.28–1.54)	<.001

CI = confidence interval, OR = odds ratio.

Table S1, Supplemental Digital Content, http://links.lww.com/MD/N193 shows a multivariable logistic regression analysis of factors associated with each domain of financial hardship. Patients who were older, male, had income equal to or more than 200% of the poverty line, and had comorbidities were less likely to experience different domains of financial hardship, while those who were uninsured were more likely to experience financial hardship. Further, participants who were of non-Hispanic Black racial or ethnic background were more likely to experience financial worry and material hardship compared to White participants and those with public insurance were less likely to experience financial worry and cost-related care nonadherence compared to those with commercial insurance.

#### 3.2.2. Psychological burden

A total of 14% reported their perceived general health status as fair or poor, 5% reported dissatisfaction with life, and 4% had SPD. Controlling for demographic factors and number of comorbidities, adults experiencing medical financial hardship had lower perceived health status (OR, 0.39; 95% CI, 0.29–0.53), lower satisfaction with life (OR, 0.37; 95% CI, 0.27–0.52), and higher odds of experiencing SPD (OR, 3.58; 95% CI, 2.43–5.47) (Table 3).

**Table 3:** Multivariable analysis* of correlation between medical financial hardship and psychological burden and moderating effect of social support and mental health therapy.

	Perceived health status OR (95% CI)	Satisfaction with life OR (95% CI)	Serious psychological distress‡ OR (95% CI)
Medical financial hardship	**0.39 (0.29–0.53**)	**0.37 (0.27–0.52**)	**3.58 (2.43–5.47**)
Frequent receipt of social or emotional support	**1.36 (1.06–1.74**)	**3.78 (2.84–5.04**)	**0.28 (0.19–0.42**)
Receipt of counseling/therapy from mental health professional in the past 12m (mental health)	**0.49 (0.40–0.60**)	**0.33 (0.25–0.43**)	**9.75 (6.97–13.94**)
Medical financial hardship * frequent receipt of social or emotional support†	1.24 (0.92–1.68)	1.22 (0.86–1.74)	0.98 (0.62–1.54)
Medical financial hardship * receipt of mental health in the last 12 months†	1.08 (0.84–1.41)	0.90 (0.64–1.25)	**0.57 (0.39–0.85**)

CI = confidence interval, OR = odds ratio.

Bold represents *P* value < .05.

Bold indicates significance.

* Adjusted for age, sex, race, income, insurance, and number of comorbidities.

† Denotes interaction between medical financial hardship domain and social support or mental health.

‡ Indicated Kessler-6 score of 13 or more.

#### 3.2.3. Social and mental health support

Overall, 11% had received counseling/therapy from mental health professionals in the past 12 months, and 90% had experienced frequent social or emotional support (Table 1).

Frequent receipt of social or emotional support was associated with lower probability of experiencing medical financial hardship (OR, 0.71; 95% CI: 0.63–0.80) (Table 2 and Table S1, Supplemental Digital Content, http://links.lww.com/MD/N193), and serious distress (OR, 0.28; 95% CI, 0.19–0.42), as well as higher probability of improved perceived health status (OR, 1.36; 95% CI, 1.06–1.74) or satisfaction with life (OR, 3.78, 95% CI, 2.84–5.04). However, the relationship between medical financial hardship and psychological burden did not significantly differ as a function of social support frequency (Table 3).

Receipt of counseling or therapy from mental health professionals in the past 12 months was associated with higher probability of experiencing medical financial hardship (OR, 1.40; 95% CI, 1.28–1.54) (Table 2) and worse psychological burden including worse perceived health status (OR, 0.49; 95% CI, 0.40–0.60), and satisfaction with life (OR, 0.33; 95% CI, 0.25–0.43) and increased serious distress (OR, 9.75; 95% CI, 6.97–13.94) compared to those not receiving mental support (Table 3). Those experiencing medical financial hardship had lower odds of developing SPD if they received mental healthcare in the last 12 months (OR, 0.57; 95% CI, 0.39–0.85).

## 4. Discussion

Using a U.S. nationally representative survey, we found that nearly half of the adult U.S. population (approximately 125 million) experienced medical financial hardship in 2021. Financial worry was the most common manifestation of medical financial hardship (44.6%), followed by cost-related care nonadherence (14.2%) and material hardship (10.5%). Our study further showed that medical financial hardship is associated with psychological burden, and social support is associated with improvement in both medical financial hardship and psychological burden. Lastly, receipt of professional mental health counseling among those who experience medical financial hardship is associated with significant decrease in serious distress when compared to those who do not experience medical financial hardship.

Our results were consistent with previous literature, suggesting medical financial hardship is a common issue among adult Americans.^[17–21]^ The sociodemographic factors associated with medical financial hardship in the current study were also concordant with prior studies, with younger age, female, racial/ethnic minorities, low income, and uninsured population as well as those with a higher number of comorbidities being at higher risk of experiencing medical financial hardship.^[18,22,23]^ Further, those with commercial insurance had higher odds of experiencing medical financial hardship compared to those with public insurance, likely due to the higher burden of cost-sharing in the underinsured population and the high prevalence of high deductible insurance plans.^[24,25]^

Our study also showed that medical financial hardship is associated with psychological burden which highlights the need for a variety of behavioral interventions to mitigate medical financial hardship.^[12]^ These include financial education,^[26–28]^ patient and/or financial navigation,^[27,29–32]^ informational decision support,^[33]^ and out-of-pocket payment elimination.^[34–36]^ For early intervention, healthcare providers and social workers should work together. Healthcare providers have a unique opportunity to assess medical financial hardship due to their established relationship with the patient, but often have limited time with their patients which makes it challenging to address medical financial hardship.^[37]^ In the majority of U.S. health systems patients identified as high risk for financial hardship will be referred to social workers who are dedicated staff with the expertise to lead financial counseling and navigation, help patients with insurance optimization and applications for financial assistance, and facilitate delivery of high-quality care.^[14,38]^ Early connection with social workers will also help establish trust between patient and health system, resulting in patients feeling safe reporting medical financial hardship.^[14,39]^

Our study’s significance lies in reporting the moderating role of social support and mental health support on medical financial hardship and psychological burden which the current literature lacks. Our study suggests that social or emotional support on its own can be used as an intervention to alleviate medical financial hardship. Further, frequent social support may alleviate psychological burden among those experiencing medical financial hardship, consistent with prior studies,^[40]^ but also among those not experiencing medical financial hardship. Our results are consistent with prior research that those with access to robust social support networks experience greater resiliency, less psychological distress, and overall enhanced well-being.^[41]^ Social workers play an important role in enhancing patients’ social support networks, by increasing access to financial and emotional resources,^[42]^ especially for patients who are nonadherent to recommended care secondary to medical financial hardship.^[14]^ The impact of specific interventions provided as well as the context in which services are delivered by clinical social work staff (i.e., coping strategies/techniques, referrals to community resources, providing support groups, and education), determines how the patient improves psychologically, which in turn will determine how the patient reacts to and perceives their burden.^[14,39]^

While prior receipt of counseling or therapy from a mental health professional is a sign of the severity of psychological burden as well as medical financial hardship, our study further showed those experiencing medical financial hardship had lower odds of psychological burden if they received mental health support, which emphasizes the importance of early screening for medical financial hardship and early intervention to mitigate consequences of medical financial hardship. Additionally, monitoring patients throughout the treatment is vital, as individuals who have long-term mental health problems before treatment and experience medical financial hardship are more likely to encounter mental health issues in the future if continued support is not offered. This study adds to evidence that mental health care might help adults with medical financial hardship to alleviate their psychological burden.

Our study has clinical implications. It is essential for healthcare providers and social workers to be familiar with screening tools to ensure individuals’ access to the assistance they need to reduce medical financial hardships as they continue their treatment.^[14]^ Future interventions should also focus on improving social support and mental health for patients as a way of mitigating medical financial hardship. With the increase in awareness for mental health care post COVID-19 pandemic, more studies are needed to support increased funding to provide mental health care to patients who are experiencing medical financial hardship. Further research regarding specific impacts to diverse populations (such as Hispanic and non-Hispanic Black population) is warranted to determine the impact of interventions efficacy in this population.

One of the strengths of our study is using a nationally representative database which will help with the generalizability of the results. However, our study has several limitations. Due to the nature of being a retrospective analysis of a national survey, the causal relationship between the association of medical financial hardship and the psychological burden was not studied. Further, domains of medical financial hardship in different studies are defined using different self-reported questions resulting in some variability in reported prevalence. Given no individual employment status is reported in NHIS, we could not assess the impact on medical financial hardship and psychological burden.

## 5. Conclusion

Experience of medical financial hardship in the United States were associated with higher degrees of psychological symptom burdens such as lower perceived health status, lower satisfaction with life, and higher odds of SPD, even after adjusting for receipt of mental health or social health support. However, social health support was associated with improvement of medical financial hardship and its psychological burden. Further, while mental health support is not associated with decreased medical financial hardship, it was associated with improved SPD among those experiencing medical financial hardship.

## Author contributions

**Conceptualization:** Gelareh Sadigh.

**Formal analysis:** Kumar Mukherjee, Gelareh Sadigh.

**Investigation:** Jinho Jung, Kumar Mukherjee, Gelareh Sadigh.

**Methodology:** Gelareh Sadigh.

**Validation:** Gelareh Sadigh.

**Writing – original draft:** Jinho Jung.

**Writing – review & editing:** Kumar Mukherjee, Mary Brown, Gelareh Sadigh.

## Supplementary Material


