# Supplementary material for: Association between financial hardship and psychological burden and the role of social and mental health support: An observational study
Source: Medicine (Baltimore). 2024 Jul 12;103(28):e38871. doi: 10.1097/MD.0000000000038871 (PMC11245238; doi:10.1097/MD.0000000000038871)
Supplement: Supplementary file 1 [file medi-103-e38871-s001.docx]

**Association between financial hardship and psychological burden and the role of social and mental health support**

**Supplementary Table 1. Multivariable logistic regression analysis of factors associated with domains of financial hardship.** Bold represents p value <0.05.

| **Factors** | **Financial Worry**  **OR (95% CI)** | **Material Hardship**  **OR (95% CI)** | **Cost-related Care nonadherence**  **OR (95% CI)** |
| --- | --- | --- | --- |
| **Age** | **0.99 (0.99-0.99)** | **0.99 (0.98-0.99)** | **0.98 (0.98-0.98)** |
| **Gender** |  |  |  |
| Female | Reference | Reference | Reference |
| Male | **0.76 (0.72-0.81)** | **0.74 (0.66-0.82)** | **0.65 (0.59-0.70)** |
| **Race** |  |  |  |
| Non-Hispanic white only | Reference | Reference | Reference |
| Non-Hispanic Black/African American only | **1.17 (1.05-1.30)** | **1.48 (1.28-1.70)** | 0.90 (0.78-1.04) |
| Hispanic | **1.73 (1.58-1.91)** | 1.06 (0.90-1.25) | **0.79 (0.69-0.90)** |
| Others | **1.30 (1.16-1.46)** | 0.84 (0.69-1.04) | **0.72 (0.61-0.84)** |
| **Income** |  |  |  |
| Income < 200% of poverty line | Reference | Reference | Reference |
| Income ≥ 200% of poverty line | **0.60 (0.56-0.66)** | **0.46 (0.41-0.52)** | **0.65 (0.58-0.72)** |
| **Insurance** |  |  |  |
| Commercial | Reference | Reference | Reference |
| Medicare | **0.68 (0.62-0.75)** | 0.86 (0.72-1.02) | **0.79 (0.68-0.92)** |
| Medicaid | **0.67 (0.60-0.75)** | 0.82 (0.67-1.00) | **0.77 (0.65-0.90)** |
| Other insurance | **0.67 (0.59-0.78)** | **1.39 (1.83-2.63)** | 1.05 (0.86-1.27) |
| Uninsured | **3.10 (2.72-3.53)** | **2.20 (1.85-2.63)** | **3.38 (3.04-3.98)** |
| **Number of comorbidities** |  |  |  |
| 0 | Reference | Reference | Reference |
| 1 | **1.25 (1.15-1.34)** | **1.75 (1.52-2.02)** | **1.72 (1.54-1.92)** |
| ≥ 2 | **1.39 (1.29-1.51)** | **2.72 (2.35-3.14)** | **2.62 (2.31-2.97)** |
| Frequent receipt of social or emotional Support | **0.74 (0.66-0.84)** | **0.68 (0.58-0.79)** | **0.59 (0.51-0.68)** |
| Receipt of counseling/therapy from mental health professional in the past 12m | **1.23 (1.12-1.36)** | **1.49 (1.30-1.71)** | **2.33 (2.08-2.62)** |

OR, Odds Ratio; CI, Confidence Interval.
